# 

**Published:** 1913-05-31

**Authors:** 


### ANSWERS TO CORRESPONDENTS.

Nurse D. E. writes that she is away from home, and desires to know to whom she should apply for medical treatment.

She should apply by letter to the clerk to the Insurance Committee for her *home* address. A green ticket will then be furnished, which, on presentation to a panel doctor in the district in which she is temporarily resident, will entitle her to treatment.

Lists of panel doctors and of the addresses of clerks to Insurance Committees can be consulted at the local post office.

### Personalia.

Dr. William Leslie Burgess has been appointed tuberculosis officer for Dundee.

The Trustees of the Hunterian Museum at the Royal College of Surgeons have elected Lord Morley of Blackburn and Sir John Wolfe Barry trustees of the collection.

Lady St. Helier has resigned her membership of the Bexley Mental Hospital Sub-Committee of the London County Council, and has been appointed a member of the Hanwell Mental Hospital Sub-Committee.

At the anniversary meeting of the Royal Geographical Society on Monday last, when Lady Scott was presented with a casket containing the Patrons' and Antarctic Medals awarded to her late husband in 1904, an award was also made of the Patrons' Medal intended for Dr. E. A. Wilson, who died with Captain Scott.

The London County Council have decided to grant a personal allowance of £50 a year, in addition to his salary of £400, to Mr. W. S. Toynbee, the settlement officer of the Mental Hospitals Committee. The Council were informed that Mr. Toynbee has been most successful in effecting saving to the Council in connection with patients chargeable to the county.

Dr. M. M. Khan, late house physician at the Brompton Hospital for Consumption, has been appointed medical officer in charge of the special tuberculosis department which has been organised at University College Hospital. A consultation concerning suspected cases and, as far as possible, the organisation of after-care for discharged patients will be undertaken.

Dame Eleanor Mary Laking, wife of Sir Francis Laking, Physician in Ordinary to the King, daughter of Mr. James Hackworth, of Rosslyn, Dunedin, and widow of Mr. Julius Angerstein, left estate valued at £1,784 gross, with net personality £329.

### Obituary.

Deputy-Inspector-General William Rogerson White, C.B., R.N., has died at the age of sixty-two. Entering the Navy as a surgeon in 1871, he took part, as fleet surgeon, in the punitive expedition against Fodi Silah, a slave-trader, on the West Coast of Africa. His services were rewarded with the C.B. In 1905 he retired with the rank of Deputy-Inspector-General of Hospitals and Fleets.

### Appointments.

Mr. R. Warrenne Dale Hewson, L.R.C.P. & S. (Edin.), L.F.P. & S. (Glas.), has been appointed assistant medical officer to Coton Hill Mental Hospital, Stafford.

Dr. J. J. Buchan, medical officer of health at St. Helens, has been recommended by the health committee to the post of medical officer of Bradford at a salary of £800 a year.

The following appointments at the Manchester Royal Infirmary have been confirmed by the board of management:—House Physicians: Mr. Harold Heathcote, M.B., Ch.B.(Vict.), Mr. J. E. Rivera, M.R.C.S., L.R.C.P.; Senior House Surgeons: Mr. R. P. Stewart, M.B., Ch.B.(Vict.), M.R.C.S., L.R.C.P., Mr. K. D. Bean, M.B., Ch.B.(Vict.); Junior House Surgeon: Mr. H. D. Willis, L.M.S.S.A.(Lond.); House Surgeon to Special Departments: Mr. T. H. Oliver, B.A., B.C.(Cantab.), M.B., Ch.B.(Vict.).

